# Experimental and Natural Induction of *de novo* Centriole Formation

**DOI:** 10.3389/fcell.2022.861864

**Published:** 2022-04-04

**Authors:** Kasuga Takumi, Daiju Kitagawa

**Affiliations:** Department of Physiological Chemistry, Graduate School of Pharmaceutical Science, The University of Tokyo, Tokyo, Japan

**Keywords:** centrosome, centriole, *de novo* centriole formation, PLK4, multicilia

## Abstract

In cycling cells, new centrioles are assembled in the vicinity of pre-existing centrioles. Although this canonical centriole duplication is a tightly regulated process in animal cells, centrioles can also form in the absence of pre-existing centrioles; this process is termed *de novo* centriole formation. *De novo* centriole formation is triggered by the removal of all pre-existing centrioles in the cell in various manners. Moreover, overexpression of polo-like kinase 4 (Plk4), a master regulatory kinase for centriole biogenesis, can induce *de novo* centriole formation in some cell types. Under these conditions, structurally and functionally normal centrioles can be formed *de novo*. While *de novo* centriole formation is normally suppressed in cells with intact centrioles, depletion of certain suppressor proteins leads to the ectopic formation of centriole-related protein aggregates in the cytoplasm. It has been shown that *de novo* centriole formation also occurs naturally in some species. For instance, during the multiciliogenesis of vertebrate epithelial cells, massive *de novo* centriole amplification occurs to form numerous motile cilia. In this review, we summarize the previous findings on *de novo* centriole formation, particularly under experimental conditions, and discuss its regulatory mechanisms.

## Introduction

Centrioles are organelles that organize centrosomes and cilia. They are cylindrical structures with a nine-fold radial symmetry of triplet or doublet microtubules. The centrosome, which consists of the centriole and the pericentriolar material (PCM)—a matrix of proteins surrounding the centriole—acts as a microtubule-organizing center (MTOC) in the cell. In addition, it plays a pivotal role in the formation of the mitotic spindle, subsequent chromosome segregation, and cytokinesis. The centriole also serves as a basal body in the formation of cilia and flagella.

**FIGURE 1:**
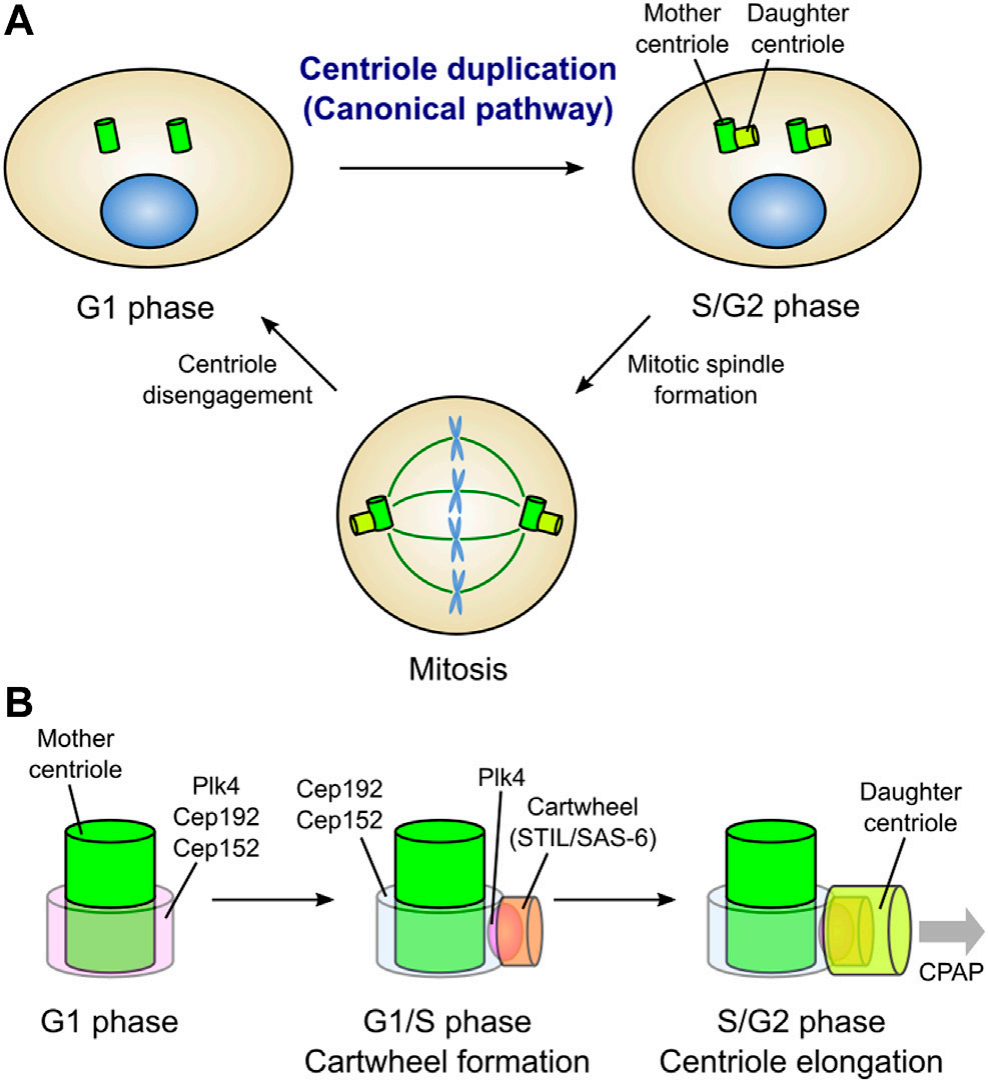
Canonical centriole duplication cycle. **(A)** Centriole duplication cycle in animal somatic cells. Canonical centriole duplication begins at the G1/S transition. New centrioles (daughter centrioles) are formed from the proximal end of the pre-existing centrioles (mother centrioles). Each pair of mother and daughter centrioles acts as the core of a single centrosome. Two centrosomes function as bipolar spindle poles in mitosis. At the mitotic exit, daughter centrioles are disengaged from the mother centrioles and convert into functional centrosomes. Thus, just two centrosomes always exist in a cell. **(B)** Evolutionarily conserved proteins involved in canonical centriole duplication in vertebrate cells. In the G1 phase, Plk4, a master kinase for centriole biogenesis, localizes in a ring-like pattern at the proximal end of the mother centriole along with Cep192 and Cep152, which cooperatively recruit Plk4. At the G1/S transition, Plk4 is re-distributed on a single focus around the mother centriole. Then, Plk4 binds to and phosphorylates STIL, facilitating STIL/SAS-6 interaction. SAS-6 in turn self-assembles to form a cartwheel structure, the basis for centriole assembly, perpendicularly to the mother centriole wall. Following the cartwheel formation, CPAP and other centriolar proteins promote the elongation of the daughter centriole.

In cycling somatic cells, new centrioles (daughter centrioles) are formed at the proximal end of pre-existing centrioles (mother centrioles). This process is referred to as “centriole duplication”, and occurs only once during the cell cycle; only one daughter centriole is formed from its mother centriole. At the end of mitosis, the daughter centrioles are disengaged from the mother centrioles and are converted into functional centrosomes, acquiring the ability to assemble new centrioles in the daughter cells. In this way, the number of centrosomes in a cell is constantly maintained at two (Figure 1A) (Loncarek and Bettencourt-Dias, 2018; Nigg and Holland, 2018).

In the context of canonical centriole duplication, the three proteins, polo-like kinase 4 (Plk4), SCL/TAL1 interrupting locus (STIL), and spindle assembly 6 homolog (SAS-6), have been identified as conserved essential factors for daughter centriole assembly (Figure 1B) (Arquint and Nigg, 2016). Loss of either of these proteins inhibits centriole duplication, while their overexpression results in the formation of multiple daughter centrioles from a single mother centriole (overduplication). Plk4 (Plk4 or Sak in *Drosophila* and ZYG-1 in *Caenorhabditis elegans*) is a serine/threonine kinase that localizes around the mother centriole and acts as a master regulator of centriole biogenesis (Bettencourt-Dias et al., 2005; Habedanck et al., 2005; Kleylein-Sohn et al., 2007). Plk4 directly binds to and phosphorylates STIL (anastral spindle 2 [Ana2] in *Drosophila* and SAS-5 in *Caenorhabditis elegans*) during the G1/S phase, thereby facilitating the STIL/SAS-6 interaction and the formation of a cartwheel structure (the basis for centriole assembly) (Dzhindzhev et al., 2014; Ohta et al., 2014; Arquint et al., 2015; Kratz et al., 2015; Moyer et al., 2015). SAS-6 is a component of the cartwheel structure, and its self-assembly is a basis for the nine-fold symmetric structure of the centriole (Nakazawa et al., 2007; Kitagawa et al., 2011; van Breugel et al., 2011).

Plk4 localizes in a ring around the mother centriole prior to the assembly of daughter centrioles in the G1 phase. In invertebrate cells, spindle defective 2 (Spd-2; centrosomal protein 192 [Cep192] in human) is responsible for the centriolar localization of ZYG-1 in *Caenorhabditis elegans*, while asterless (Asl; Cep152 in human) is responsible for the centriolar localization of Plk4/Sak in *Drosophila* (Delattre et al., 2006; Pelletier et al., 2006; Dzhindzhev et al., 2010). In mammalian cells, Cep192 and Cep152, which localize in a ring around the mother centriole, cooperatively recruit Plk4 to the centriole (Kim et al., 2013; Sonnen et al., 2013). Following the G1/S transition, Plk4 is distributed on a single focus around the mother centriole, and co-localizes with STIL/SAS-6, leading to the formation of the cartwheel structure (Figure 1B) (Kim et al., 2013; Ohta et al., 2014).

After cartwheel formation, centrosomal P4.1-associated protein (CPAP; SAS-4 in *Drosophila* and *Caenorhabditis elegans*) facilitates the formation of the centriole microtubule wall (Pelletier et al., 2006; Kohlmaier et al., 2009; Schmidt et al., 2009; Tang et al., 2009). Cep135 (Bld10 in *Drosophila*) seems to connect SAS-6 with CPAP for the stabilization and elongation of the centriole wall (Lin Y.-C. et al., 2013). Centriolar proteins including Cep120 (Lin Y.-N. et al., 2013; Comartin et al., 2013), Centrobin (Gudi et al., 2011, 2015), POC1 (Keller et al., 2009) and Cep295 (Ana1 in *Drosophila*) (Chang et al., 2016; Saurya et al., 2016), also positively regulate centriole elongation. In contrast, the centriolar coiled-coil protein 110 (CP110)/Cep97 protein complex acts as a cap at the distal end of centrioles to restrict centriole elongation in human cells (Spektor et al., 2007; Kohlmaier et al., 2009; Schmidt et al., 2009; Tang et al., 2009).

It has also been established that numerous species utilize an alternative pathway for centriole biogenesis, which is driven without pre-existing centrioles in the cell (*de novo* pathway). In addition to its occurrence under physiological conditions in various species, *de novo* centriole formation can be induced artificially under experimental conditions in eukaryotic cells. While centriole duplication via the canonical pathway has been extensively studied in recent years, the mechanisms regulating the *de novo* pathway remain largely unexplored. In this review, we will summarize the findings of recent studies on *de novo* centriole formation under experimental conditions and discuss the regulatory mechanisms of the *de novo* pathway in comparison with the canonical pathway.

## *De novo* Centriole Formation Following Removal of the Resident Centrioles

**FIGURE 2:**
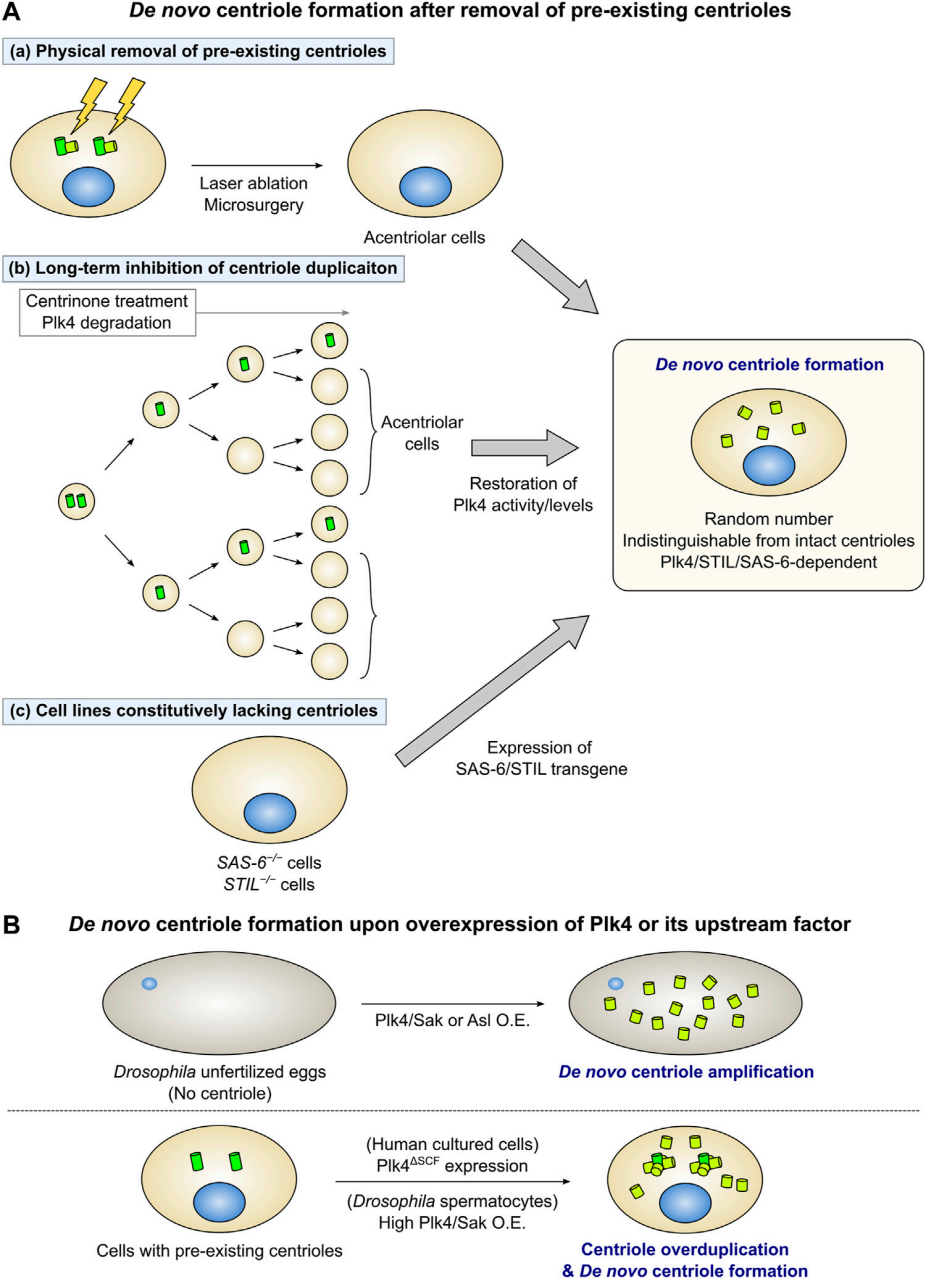
*De novo* centriole formation under experimental conditions. **(A)** Removal of all the pre-existing centrioles triggers *de novo* centriole formation. **(a)** When all the pre-existing centrioles are physically removed by laser ablation or microsurgery, new centrioles are formed *de novo* in the cytoplasm. **(b)** Long-term inhibition of centriole duplication by chronic treatment with centrinone (a Plk4 inhibitor) or Plk4 protein degradation decreases the number of centrioles in cycling cells. After the cells lose all centrioles, the restoration of Plk4 activity or levels leads to *de novo* centriole formation. **(c)** STIL/SAS-6 transgene expression in STIL/SAS-6 knockout acentriolar cells triggers *de novo* centriole formation. In each case, a random number of centrioles are formed *de novo*. Many of the centrioles formed *de novo* are structurally and functionally indistinguishable from intact centrioles. Additionally, the *de novo* pathway depends on Plk4, STIL, and SAS-6 as well as the canonical pathway does. **(B)** Overexpression of Plk4 or its upstream factor can induce *de novo* centriole formation. (Upper) In *Drosophila* unfertilized eggs, which do not have a centriole, overexpression of Plk4/Sak or Asl induces *de novo* centriole amplification. (Lower) Expression of Plk4^ΔSCF^ (an undegradable mutant of Plk4) in human cultured cells leads to *de novo* centriole formation, in addition to centriole overduplication from the pre-existing centrioles. High levels of Plk4/Sak overexpression in *Drosophila* primary spermatocytes also induce *de novo* formation and overduplication of centrioles.

In animal somatic cells with centrioles, new centrioles can be formed through the *de novo* pathway following the removal of all resident centrioles (Figure 2A). In early studies, this phenomenon was observed in *Chlamydomonas* cells (Marshall et al., 2001). Marshall et al. used a mutant with defective centriole segregation to generate acentriolar cells, and found that new centrioles were formed *de novo* in those cells. In Chinese hamster ovary cells arrested in the S phase, physical removal of all centrioles by laser ablation induced *de novo* centriole formation. After removing the centrioles, foci containing the PCM proteins γ-tubulin and Pericentrin (PCM cloud) initially appeared. This was followed by the appearance of centrioles with a normal ultrastructure within the PCM cloud (Khodjakov et al., 2002). It has been reported that *de novo* centriole formation can occur similarly in human cultured cells. Human cells in which all centrioles had been removed by laser ablation or microsurgery assembled new centrioles *de novo* in the S phase (Figure 2Aa) (La Terra et al., 2005; Uetake et al., 2007). Notably, *de novo* centriole formation was suppressed as far as at least one centriole remained in the cell (La Terra et al., 2005).

Several studies demonstrated *de novo* centriole formation through the generation of acentriolar cells by genetic manipulation or drug treatment. Centrinone, a selective and reversible inhibitor of Plk4 enables the easy removal of centrioles in various mammalian cell types. Inhibition of centriole duplication via long-term treatment with centrinone generates cell populations that are predominantly free of centrioles. Subsequent washout of centrinone can induce *de novo* centriole formation by restoring the activity of Plk4 (Figure 2Ab) (Wong et al., 2015). *De novo* centriole formation can also be observed using a system that induces reversible degradation of the Plk4 protein. Introducing the plant-derived, auxin-inducible degron system into mammalian cells enables the rapid degradation of target proteins by treatment with the plant hormone auxin [indole-3-acetic acid (IAA)] (Nishimura et al., 2009). Following the addition of IAA to cells with an auxin-inducible degron-tagged, endogenous Plk4 gene for several days to induce the degradation of the Plk4 protein, the number of centrioles in the cell was found to decrease with each cell cycle. After the complete loss of centrioles, restoration of Plk4 protein levels by IAA washout led to *de novo* biogenesis of new centrioles in the cytoplasm (Figure 2Ab) (Lambrus et al., 2015). Similarly, *de novo* centriole formation induced by the removal of resident centrioles has been observed in cultured cell lines of *Drosophila melanogaster*. Loss of centrioles by long-term treatment with Plk4/Sak RNAi, followed by the restoration of Plk4/Sak expression, results in the *de novo* assembly of new centrioles in *Drosophila* cell lines (Rodrigues-Martins et al., 2007b; Dzhindzhev et al., 2010; Nabais et al., 2021).

Cell lines in which essential factors for centriole duplication, such as SAS-6 and STIL, are knocked out, do not have centrioles. Wang et al. established an experimental system to observe *de novo* centriole formation by inducing the expression of the SAS-6 transgene in *SAS-6* ^*−/−*^ cell lines (Wang et al., 2015). Similarly, expressing the STIL transgene in STIL-knockout human cell lines or mouse embryonic fibroblasts can induce *de novo* centriole formation (Figure 2Ac) (Castiel et al., 2011; David et al., 2014; Liu et al., 2018).

Depleting Cep295 prevents newly formed centrioles from acquiring the ability to duplicate, and also destabilizes their structure (Izquierdo et al., 2014). Cells lacking the tubulin family members delta-tubulin or epsilon-tubulin generate unstable centrioles that lack triplet microtubules (Wang et al., 2017). Long-term loss of these factors leads to degradation of the unstable centrioles during mitosis and continuous centriole duplication failure, giving rise to acentriolar cells. Many of these acentriolar cells assemble centrioles *de novo* during the S phase. These centrioles formed through the *de novo* pathway are also unstable and disappear after mitosis (Izquierdo et al., 2014; Wang et al., 2017).

As above, *de novo* centriole formation can be induced by removing resident centrioles through various approaches. The number of newly-formed centrioles through the *de novo* pathway is random in the first cycle, thus suggesting that cells do not possess a system for maintaining the strict control of this number, unlike in canonical centriole duplication (Khodjakov et al., 2002; La Terra et al., 2005; Lambrus et al., 2015; Wong et al., 2015). After *de novo* centriole formation takes place, the number of centrioles in the cell gradually returns to normal values (Wong et al., 2015). This may be partly due to the death of cells with an excessive number of centrosomes through abnormal mitosis. Studies using electron microscopy (EM) revealed that *de novo* centriole formation is prone to ultrastructural errors (Khodjakov et al., 2002; Wang et al., 2015), while some of the centrioles formed *de novo* appear to be equivalent to intact centrioles with triplet microtubules (Khodjakov et al., 2002; La Terra et al., 2005; Uetake et al., 2007; Lambrus et al., 2015; Wang et al., 2015). Centriole/centrosome proteins Plk4, STIL, SAS-6, CPAP, Centrin, Cep135, Cep152, Cep192, Pericentrin, Cep164, and centrosomal Nek2-associated protein 1 (Cep250/C-Nap1) are recruited to the centrioles formed *de novo*. This finding suggested that they are indistinguishable from intact centrioles in terms of their main components (Lambrus et al., 2015). Moreover, the centrioles formed *de novo* can duplicate and recruit PCM to form microtubule asters, indicating that they have the ability to grow as functional mother centrioles (La Terra et al., 2005; Lambrus et al., 2015). Regarding the relationship with the cell cycle, *de novo* centriole formation in proliferating cells occurs after entry into the S phase, but not in the G1 phase (Khodjakov et al., 2002; La Terra et al., 2005; Izquierdo et al., 2014; Lambrus et al., 2015; Wang et al., 2017), which is consistent with canonical centriole duplication.

## Molecules Required for *de novo* Centriole Formation

Do common molecules function in the canonical and *de novo* pathway for centriole assembly? Prolonged depletion of Plk4 or inhibition of its kinase activity produces acentriolar cells by blocking centriole duplication. In these conditions, centrioles do not assemble *de novo* unless active Plk4 levels are restored (Rodrigues-Martins et al., 2007b; Lambrus et al., 2015; Wong et al., 2015). These findings indicate that the activity of Plk4 is essential for centriole assembly through the *de novo* pathway as well. STIL (Castiel et al., 2011; David et al., 2014; Lambrus et al., 2015; Liu et al., 2018) and SAS-6 (Wang et al., 2015) are also essential for *de novo* centriole biogenesis in mammalian cells, as depletion of these proteins blocks the formation of centrioles by both canonical and *de novo* pathways. SAS-6 and SAS-4 are required for *de novo* centrosome assembly as in the canonical pathway in cultured *Drosophila* cells (Rodrigues-Martins et al., 2007b). These results indicate that the core factors behind the formation of the structure of the centriole are common regardless of whether a new centriole is formed in association with the mother centriole or not. It has also been shown that the interaction between these core factors is similarly critical for *de novo* centriole formation. For example, in the case of STIL, its oligomerization via its coiled-coil domain (David et al., 2016), phosphorylation by Plk4 at multiple sites, and phosphorylation-mediated interaction with SAS-6 and CPAP (Moyer and Holland, 2019), appear to be required for both canonical and *de novo* centriole formation in mammalian cells. However, it has been suggested that the *de novo* pathway may not require oligomerization of SAS-6 (Wang et al., 2015), meaning that there may be some mechanisms of centriole formation in the *de novo* pathway that differ from those observed in the canonical pathway.

In cultured *Drosophila* cells, the Plk4/Sak recruiter protein Asl is required for *de novo* centriole formation after the removal of resident centrioles (Dzhindzhev et al., 2010; Nabais et al., 2021). This finding suggests that the cytoplasmic Asl somehow contributes to *de novo* centriole formation through the local accumulation of Plk4/Sak; however, the underlying mechanisms remain unclear. It will also be necessary to investigate whether the upstream factors of Plk4 promote *de novo* centriole formation in mammalian cells. Other PCM proteins (Pericentrin-like protein [Plp], centrosomin [Cnn], Spd-2, and γ-tubulin) also support *de novo* centriole formation, at least to some extent, in *Drosophila* cells. In particular, the contribution of γ-tubulin is relatively significant, as its depletion attenuates *de novo* centriole assembly in both cultured cells and unfertilized eggs of *Drosophila* (Nabais et al., 2021). The observation that centrioles are formed *de novo* in the PCM cloud containing γ-tubulin and Pericentrin in Chinese hamster ovary cells (Khodjakov et al., 2002) implies that PCM proteins are implicated in the *de novo* pathway prior to centriole assembly. Considering that Pericentrin is involved in the recruitment of SAS-6 (Ito et al., 2019), it is possible that PCM proteins serve as a scaffold in the cytoplasm and accumulate proteins necessary for centriole biogenesis.

## Regulation of *de novo* Centriole Formation by the Local Concentration of Plk4

Overexpression of Plk4/Sak in *Drosophila* unfertilized eggs results in the formation of numerous centrioles (Figure 2B) (Peel et al., 2007; Rodrigues-Martins et al., 2007b). Since the unfertilized eggs do not originally have a centriole, these centrioles are initially formed *de novo*. EM observation confirmed that Plk4/Sak-induced centrioles are structurally normal (Rodrigues-Martins et al., 2007b). A recent study investigated a controlled system that allows for the high-resolution imaging of *de novo* centriole formation, using *Drosophila* egg explants overexpressing Plk4/Sak (Nabais et al., 2021). Asl overexpression in *Drosophila* unfertilized eggs also results in *de novo* biogenesis of centrioles with an intact ultrastructure (Figure 2B) (Dzhindzhev et al., 2010). Overexpression of SAS-4, SAS-6, Ana1, and Ana2 in *Drosophila* unfertilized eggs leads to the *de novo* formation of centriolar protein-containing aggregates (Rodrigues-Martins et al., 2007a; Peel et al., 2007; Dobbelaere et al., 2008; Stevens et al., 2010). In particular, when SAS-6 and Ana2 are co-overexpressed in *Drosophila* eggs, they form large ring-shaped structures independent of the influence of Plk4/Sak (Stevens et al., 2010; Gartenmann et al., 2020). This observation suggests that, in addition to the self-assembly property of SAS-6 and Ana2, Plk4/Sak may be necessary for their organization into a nine-fold symmetric structure as a part of the centriolar cartwheel structure.

As mentioned above, overexpression of Plk4/Sak or its loader protein Asl in *Drosophila* unfertilized eggs induces *de novo* centriole formation, implying that Plk4/Sak is particularly important as a regulator of the *de novo* pathway. Locally concentrated Plk4/Sak promotes its autoactivation by trans-phosphorylation (Lopes et al., 2015). Consistently, recent evidence has shown that the concentration of Plk4/Sak determines the onset of *de novo* centriole formation in *Drosophila* egg explants (Nabais et al., 2021). Meanwhile, in *Drosophila* primary spermatocytes with pre-existing centrioles, limited Plk4/Sak overexpression induces centriole amplification only from mother centrioles. In contrast, extensive Plk4/Sak overexpression in these cells can trigger *de novo* centriole formation (Figure 2B) (Lopes et al., 2015). These results suggested that pre-existing centrioles act as Plk4/Sak accumulators. Moreover, once the local concentration of Plk4/Sak reaches a sufficient level for the induction of the assembly of centriolar components in the cytoplasm, *de novo* centriole formation can occur even in the presence of pre-existing centrioles. Similarly, overexpression of a stable mutant of Plk4 with mutations in the degron motif (Plk4^ΔSCF^) can induce *de novo* centriole formation in human cultured cells (Figure 2B) (Wang et al., 2011). Overall, it is likely that a sufficiently high level of cytoplasmic Plk4 can trigger *de novo* centriole assembly, regardless of the presence or absence of pre-existing centrioles.

*De novo* centriole formation is normally suppressed as far as at least one centriole is present in human proliferating cells (La Terra et al., 2005; Lambrus et al., 2015). Therefore, it is expected that there is a surveillance mechanism by which the presence of a centriole in the cell suppresses ectopic centriole assembly. In other words, the cell may trigger *de novo* centriole formation only when it somehow senses the absence of centrioles. However, the mechanisms involved in triggering the *de novo* pathway remain largely unexplored. It is estimated that endogenous Plk4 levels in normal cells are very low (Bauer et al., 2016; Nabais et al., 2021). This suggests that the concentration of Plk4 is usually elevated to a sufficient level for centriole biogenesis only at the pre-existing centrioles, whereas the cytoplasmic concentration of Plk4 is controlled at very low levels to prevent ectopic centriole formation. How can Plk4 accumulate locally in the cytoplasm and reach a sufficient concentration for centriole biogenesis in acentriolar cells? Time-lapse observation of *de novo* centriole formation using a cell line expressing fluorescence-tagged Centrin (a centriole marker) has shown that Centrin foci emerge, being scattered throughout the cytoplasm (La Terra et al., 2005; Lambrus et al., 2015). These results raise the possibility that cytoplasmic Plk4 accumulates stochastically, and centrioles form *de novo* when the local concentration of Plk4 exceeds a certain threshold. However, currently, there is no evidence to reasonably explain the regulation of the local concentration of Plk4 in this manner only after the loss of centrioles. Understanding the quantitative and qualitative changes in Plk4 and its putative upstream factors, upon centriole loss, may provide insight into the mechanisms that regulate the activation and suppression of the *de novo* pathway.

## Factors Preventing Ectopic Aggregation of Centriole-Associated Proteins

**FIGURE 3:**
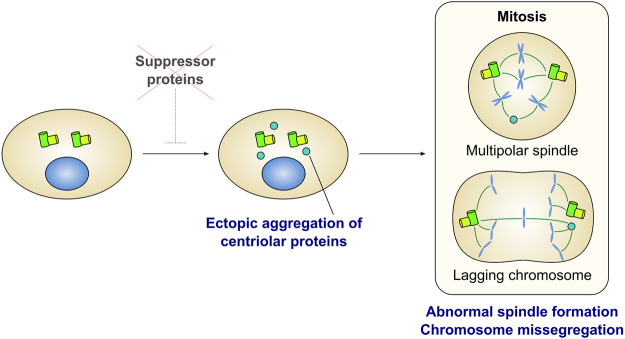
Ectopic formation of centriolar protein aggregates is detrimental for cell division. Several proteins have been identified as a suppressor of ectopic aggregation of centriole-related proteins in the cytoplasm (also see Table 1). Depletion of the suppressors leads to form ectopic aggregates containing centriolar proteins. In many cases, aggregates can act as extra spindle poles and cause mitotic defects such as multipolar spindle formation and lagging chromosomes.

Several factors prevent the aggregation of centriole-associated proteins in the cytoplasm of proliferating human cells (Figure 3 and Table 1). Depletion of Cep76 (Tsang et al., 2009), neuralized E3 ubiquitin protein ligase 4 (Neurl4) (Li et al., 2012), galectin 3 binding protein (LGALS3BP) (Fogeron et al., 2013), and RNA binding motif protein 14 (RBM14) (Shiratsuchi et al., 2015) leads to the formation of centriolar protein-containing cytoplasmic aggregates, even in the presence of intact centrosomes. These aggregates are observed by EM as electron-dense materials (Li et al., 2012; Shiratsuchi et al., 2015) or incomplete centriole-like structures with microtubules (Tsang et al., 2009; Fogeron et al., 2013; Shiratsuchi et al., 2015). These aggregates may or may not function as the major MTOC in human cells. For example, aggregates induced by the depletion of Cep76 disappear in mitosis and do not affect mitotic spindle formation (Tsang et al., 2009). In contrast, depletion of Neurl4 or RBM14 leads to the formation of structures that act as mitotic spindle poles, and results in abnormal spindle formation and defective chromosome segregation (Li et al., 2012; Shiratsuchi et al., 2015). These effects suggest that these factors play an essential role in proper chromosome segregation in mitosis (Figure 3). The composition of these cytoplasmic aggregates differs in each condition, and is often heterogeneous even within a single cell. For instance, while depletion of Neurl4 or LGALS3BP leads to the generation of centriolar protein aggregates without SAS-6 (Li et al., 2012; Fogeron et al., 2013), the assembly of the aggregates formed upon Cep76 depletion depends on SAS-6 (Tsang et al., 2009). Aggregate formation upon RBM14 depletion does not depend on SAS-6; however, some aggregates grow to become structurally similar to the centriole, presumably by incorporating SAS-6 (Shiratsuchi et al., 2015). The formation of centriole-like structures in LGALS3BP-depleted cells depends on Plk4 (Fogeron et al., 2013). On the other hand, in RBM14-depleted cells, the formation of these structures is not dependent on Plk4, but depends on the formation of the STIL-CPAP complex (Shiratsuchi et al., 2015). Depletion of Neurl4 increases the protein levels of CP110, leading to the ectopic formation of centriole-related structures (Li et al., 2012).

**TABLE 1:** Proteins that suppress ectopic aggregation of centriole-related proteins.

Suppressors	Cell types	Proteins in aggregates	Proteins not in aggregates	Proteins required for aggregation	Mitotic defects	References
*CEP76*	U2OS, Saos-2, Other osteosarcoma cell lines, Blastoma cell lines	CP110, Centrin, C-Nap1, CPAP, SAS-6, Polyglutamylated tubulin	γ-Tubulin, Pericentrin	SAS-6, CP110, Cep97	Aggregates do not persist through mitosis	Tsang et al. (2009)
*NEURL4*	U2OS, HeLa, RPE1	CP110, Centrin, γ-Tubulin, C-Nap1, Polyglutamylated tubulin	SAS-6	CP110	Pseudobipolar, Lagging chromosome	Li et al. (2012)
*LGALS3BP*	U2OS, HEK293, Human seminoma tissue	Centrin, CPAP, Acetylated tubulin, γ-Tubulin	Plk4, SAS-6, Cep135, ODF2, C-Nap1, Pericentrin, Polygltamylated tubulin	Plk4	Asterless spindle pole, Extra spindle pole	Fogeron et al. (2013)
*RBM14*	U2OS, HeLa, RPE1, NIH3T3, Mouse embryo	Centrin, Centrobin, CPAP, Acetylated tubulin, γ-Tubulin, STIL, CP110, Cep192, SAS-6	C-Nap1, Cep164	STIL, CPAP	Pseudobipolar, Multipolar, Lagging chromosome	Shiratsuchi et al. (2015)
*TRIM37* (About Centrobin-containing condensates)	HeLa, RPE1, Murlibrey nanism patients’ fibroblast	Centrobin, Plk4, SPICE, [(Mitosis) Cep192, CDK5RAP2, Pericentrin, γ-Tubulin]	SAS-6, Cep152, Many other centriole proteins	Centrobin	Pseudobipolar, Multipolar, Lagging chromosome, Micronuclei, Missegregation of chromosome 17 and 18	Balestra et al. (2021); Meitinger et al. (2020); Meitinger et al. (2021)

Recent studies have reported that ubiquitin ligase tripartite motif-containing protein 37 (TRIM37) prevents the ectopic formation of centriole protein condensates. In TRIM37-depleted cells, condensates containing Centrobin and Plk4 are observed in the cytoplasm, acquiring PCM proteins and serving as MTOCs during mitosis (Balestra et al., 2021; Meitinger et al., 2021). Centrobin is a centriolar protein that normally localizes to newly formed daughter centrioles and is involved in centriole elongation (Zou et al., 2005; Gudi et al., 2011, 2015). The assembly of the ectopic condensates in TRIM37-depleted cells depends on Centrobin, but not on Plk4. EM and super-resolution microscopy have revealed stripe patterns (Balestra et al., 2021; Meitinger et al., 2021) and hexagonally packed punctate patterns (Meitinger et al., 2021) corresponding to Centrobin condensates. Loss-of-function mutations in the TRIM37 gene cause an autosomal recessive disorder termed Mulibrey nanism (Avela et al., 2000). This genetic disorder is a kind of dwarfism with symptoms including severe growth failure, dysmorphia, and impairment in several organs. Fibroblasts from patients with Mulibrey nanism have Centrobin-containing condensates as do the cultured cell lines depleted of TRIM37 (Balestra et al., 2021; Meitinger et al., 2021). In patient-derived fibroblasts, these condensates act as MTOCs during mitosis and are accompanied by a high frequency of defects in spindle formation and chromosome segregation (Balestra et al., 2021). This observation suggests that chromosome instability due to condensate formation may be linked to the disease. Centriole dysregulation has been previously implicated in dwarfism since microcephalic primordial dwarfism, another subtype of dwarfism, is caused by mutations in several centriolar genes (Khetarpal et al., 2016; Nano and Basto, 2017). Considering this, it is plausible that abnormal condensation of centriolar proteins may be responsible for Mulibrey nanism. Besides Centrobin-containing condensates, ectopic formation of Centrobin-independent Centrin foci (Balestra et al., 2021; Meitinger et al., 2021) and Cep192 foci (Meitinger et al., 2016) in interphase has been reported in TRIM37-depleted cells. In acentriolar cells, depletion of TRIM37 leads to the formation of ectopic spindle poles with an array of cytoplasmic foci containing Cep192 and Plk4, and thereby promotes mitotic spindle assembly (Meitinger et al., 2016, 2020; Yeow et al., 2020). These results suggest that TRIM37 prevents ectopic aggregation of centriole-associated proteins in various manners.

Different assembly processes appear to underlie the aggregation of centriole-associated proteins upon depletion of each of the above factors. Thus, the mechanisms by which these factors suppress the formation of aggregates are also considered distinct. It is also conceivable that each factor only partially suppresses ectopic centriole formation through the *de novo* pathway, as the cytoplasmic aggregates observed in each case do not have the complete centriole ultrastructure. These findings imply that the suppression of *de novo* centriole formation in somatic cells is achieved through complicated mechanisms mediated by various factors; however, these mechanisms remain unclear. Considering the possibility of unidentified suppressors, future studies are warranted to comprehensively identify the factors that prevent ectopic aggregation of centriole proteins.

## *De novo* Centriole Formation Naturally Occurs in Various Species

In animal somatic cells, new centrioles are usually generated by canonical centriole duplication. Nevertheless, previous studies, mainly using EM, have revealed that several species utilize the *de novo* pathway in various manners (Nabais et al., 2017). In many species, centrioles are eliminated in oocytes and supplied from the sperm during fertilization; therefore, the unfertilized egg does not have a centriole. However, centrioles are formed *de novo* in parthenogenetic insect eggs, which initially do not have a centriole (Riparbelli et al., 1998; Riparbelli and Callaini, 2003; Ferree et al., 2006). Artificially activated eggs of sea urchins (Dirksen, 1961; Kato and Sugiyama, 1971; Miki-Noumura, 1977) and surf clams (Kuriyama et al., 1986; Palazzo et al., 1992) also form centrioles *de novo*. In rodents, the early embryo does not have a centriole because the typical centrioles or centriole-like structures have not been observed to date in the sperm. Early mouse embryos initially undergo non-centrosomal cell divisions, but centrioles appear to form *de novo* at the blastocyst stage (Szollosi et al., 1972; Gueth-Hallonet et al., 1993; Courtois et al., 2012).

Some species form centrioles *de novo* that serve as basal bodies to assemble cilia and flagella. For instance, the protist *Naegleria gruberi* uses the *de novo* pathway. *Naegleria* forms two centrioles and two flagella during its transformation from an amoeba state into a flagellate state (Dingle and Fulton, 1966; Fulton and Dingle, 1971; Fritz-Laylin et al., 2010). In this process, the first centriole is formed *de novo*, while the second one is duplicated from the first one (Fritz-Laylin et al., 2016). In plants with biflagellate sperm, such as bryophytes, as well as in the protist Labyrinthula spp., two centrioles are formed *de novo* through the “bicentriole” form. The “bicentriole” is composed of two centrioles sharing a single elongated cartwheel structure. The two centrioles subsequently separate and serve as the basal bodies forming the two flagella (Moser and Kreitner, 1970; Perkins, 1970; Robbins, 1984; Gomes Pereira et al., 2021). Similarly, multiple centrioles form *de novo* through electron-dense structures termed blepharoplasts in plants such as ferns and cycads during the generation of multiciliated sperms (Mizukami and Gall, 1966; Hepler, 1976). Planarians also form massive centrioles through the *de novo* pathway for multiciliated cells in the pharynx and body epidermis (Azimzadeh et al., 2012; Li et al., 2020).

For these examples, few studies have analyzed the molecular mechanisms that regulate *de novo* centriole biogenesis in detail; hence, these mechanisms remain largely unexplained. In the multiciliated cells of planarians, *de novo* centriole amplification requires the planarian homologs of Plk4, Cep152, CPAP, STIL and SAS-6, the conserved core proteins for centriole assembly (Azimzadeh et al., 2012; Li et al., 2020). A recent study involving the bryophyte *Physcomitrium patens* revealed that the evolutionarily conserved centriole proteins SAS-6, Bld10 (Cep135), and POC1 are required for bicentriole-mediated *de novo* centriole biogenesis (Gomes Pereira et al., 2021). These results suggest that a common molecular mechanism may be used for centriole formation in a wide range of species.

## Control of Massive Centriole Amplification in Multiciliated Cells

**FIGURE 4:**
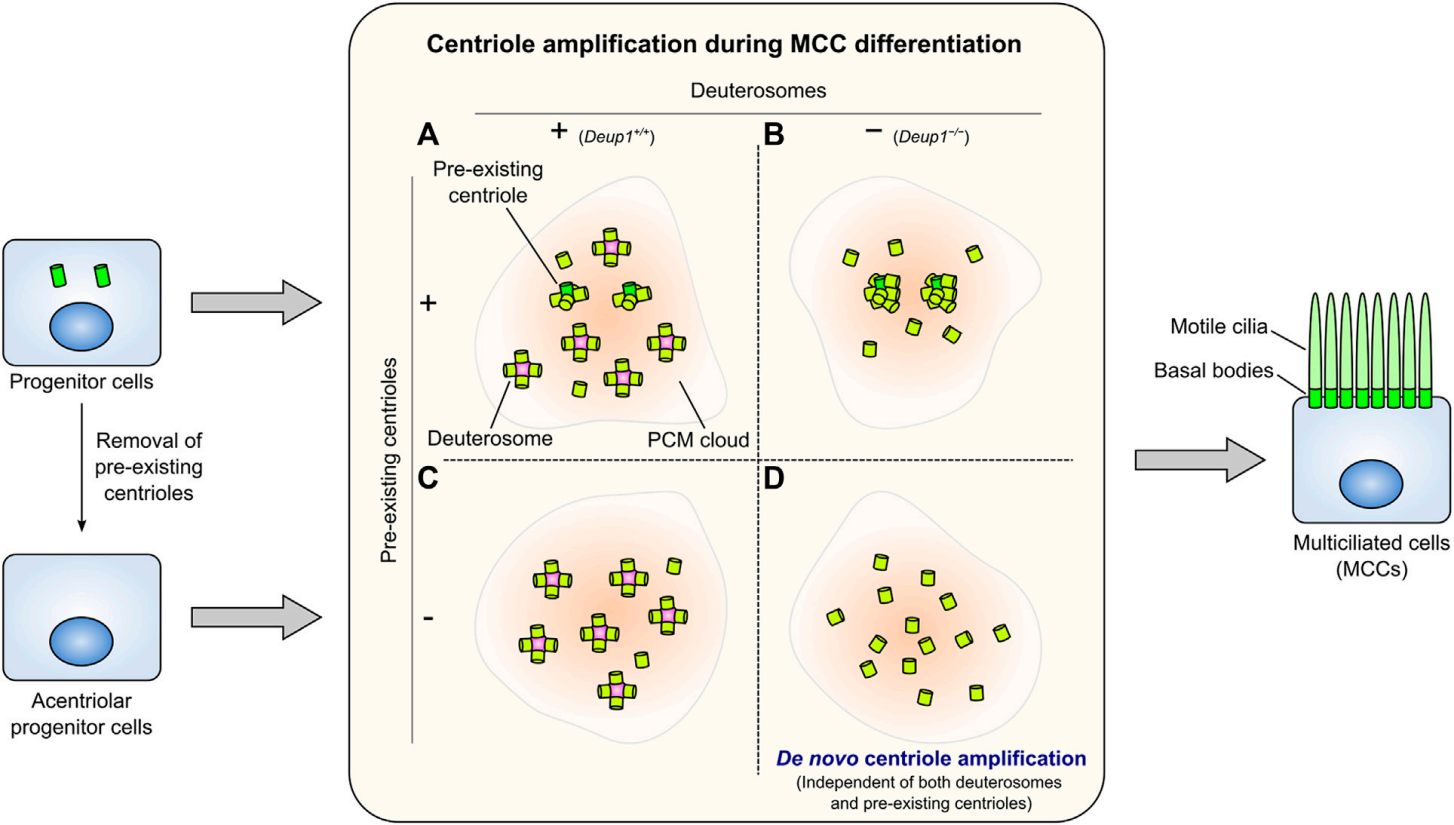
Centriole amplification in multiciliated cells (MCCs). Numerous centrioles assemble from the pre-existing centrioles and many deuterosomes in intact wild-type MCCs **(A)**. *Deup1* ^*−/−*^ MCCs, which cannot form deuterosomes, assemble more centrioles from the pre-existing centrioles **(B)**. In MCCs whose pre-existing centrioles have been removed, centrioles form predominantly through the deuterosome-dependent pathway **(C)**. MCCs can amplify the proper number of centrioles *de novo* without the pre-existing centrioles and deuterosomes **(D)**. Centriole amplification occurs presumably within a PCM cloud in each condition. These centrioles in each case dock at the plasma membrane and serve as basal bodies to form numerous motile cilia (right).

Some cells in the vertebrate airway epithelium, oviduct epithelium, and ventricular ependyma differentiate into multiciliated cells (MCCs). In MCCs, hundreds of centrioles form, serving as basal bodies to assemble numerous cilia (Figure 4). These cilia are motile and play tissue-specific roles, such as generating directional fluid flow on the luminal surface of epithelial cells. Inhibition of Notch signaling triggers changes in the transcriptional program mediated by geminin coiled-coil containing protein 1 (GEMC1) and Multicilin during MCC differentiation (Spassky and Meunier, 2017; Lewis and Stracker, 2021). EM studies revealed that MCCs generate a vast number of centrioles via scaffold structures termed deuterosomes, in addition to the centriole amplification that takes place from pre-existing centrioles (Figure 4A) (Sorokin, 1968). The deuterosome is a ring-shaped electron-dense structure that mediates the formation of multiple centrioles. Recently, research has been focused on elucidating the mechanisms that coordinate deuterosome-mediated centriole amplification (Spassky and Meunier, 2017; Boutin and Kodjabachian, 2019). The Cep63 paralogue deuterosome assembly protein 1 (Deup1) has been identified as the core component of deuterosomes (Zhao et al., 2013). Deup1 is highly expressed due to the transcriptional reprogramming during MCC differentiation. Concurrently, the expression levels of centriolar proteins (e.g., Plk4, STIL, SAS-6, Cep152, and CPAP) are also considerably increased, allowing the *de novo* formation of numerous centrioles (Vladar and Stearns, 2007; Zhao et al., 2013). Cep152 interacts with Deup1 and localizes at the deuterosomes (Zhao et al., 2013). Plk4 and SAS-6 are also recruited to the deuterosomes (Klos Dehring et al., 2013; Zhao et al., 2013), and centriole amplification depends on the latter (Vladar and Stearns, 2007). Major cell cycle regulators including cyclin-dependent kinase 2 (Cdk2), Cdk1, Plk1, Separase, and anaphase-promoting complex/cyclosome (APC/C), which are involved in centriole duplication cycle in cycling cells, also control the multi-step processes of deuterosome-mediated centriole amplification in post-mitotic MCCs (Al Jord et al., 2017; Revinski et al., 2018; Vladar et al., 2018). It has been observed that deuterosomes are derived from the vicinity of the daughter centrosome (Al Jord et al., 2014). Nevertheless, in 2019, several research groups reported that deuterosome formation and centriole amplification occur in MCCs that have lost their resident centrioles via inactivation of Plk4 (Mercey et al., 2019a; Nanjundappa et al., 2019; Zhao et al., 2019), illustrating that pre-existing centrioles are not essential for deuterosome formation (Figure 4C).

The number and morphology of basal bodies that appear during multiciliogenesis are not affected in ependymal cells derived from *Deup1* ^*−/−*^ mice, which are unable to form deuterosomes (Figure 4B). Furthermore, centriole amplification can still occur even in MCCs lacking both mother centrioles and deuterosomes (Figure 4D) (Mercey et al., 2019b). In other words, under such conditions, a massive number of centrioles form independently of mother centrioles and deuterosomes. This potentially allows us to observe phenomena similar to *de novo* centriole biogenesis in proliferating cells at a larger scale. Intriguingly, most centriole amplification in MCCs occurs in a PCM cloud containing Pericentrin; this process is also observed in the absence of deuterosomes and/or pre-existing centrioles (Mercey et al., 2019a; 2019b). This evidence suggests that PCM may play a role in *de novo* centriole formation for multiciliogenesis, independently of either pre-existing centrioles or deuterosomes. This process is reminiscent of the involvement of PCM in *de novo* centriole formation in proliferating cells (Khodjakov et al., 2002; Nabais et al., 2021). Furthermore, observation using EM has shown that the amplified centrioles are distributed in the vicinity of fibrogranular materials (FGMs) in MCCs devoid of both deuterosomes and pre-existing centrioles (Mercey et al., 2019b). Through EM, FGMs were discovered as arrays of electron-dense granules (diameter: 40–80 nm) found specifically in MCCs undergoing centriole amplification (Sorokin, 1968; Anderson and Brenner, 1971; Dirksen, 1971). A recent study revealed that FGMs contain various centriole-associated proteins and ensure the fidelity of multiciliary formation (Zhao et al., 2021). Thus, in future studies, understanding the role of PCM and FGMs in MCCs without pre-existing centrioles and/or deuterosomes may provide a better understanding of *de novo* centriole formation in proliferating cells.

## Perspective

Regarding the molecular mechanisms of centriole biogenesis, the *de novo* pathway has been studied less extensively than the canonical pathway. Further studies on the mechanisms underlying the *de novo* pathway, in conjunction with the canonical pathway, will lead to a deeper insight into the common and divergent mechanisms involved in centriole assembly. Another critical research question is the mechanism through which centriolar cells suppress the *de novo* pathway. This can be a medically significant question, since disruption of the suppression mechanisms would potentially result in uncontrolled centriole amplification and subsequent tumorigenesis (Levine et al., 2017). Therefore, detailed analysis of the molecular mechanisms regulating the *de novo* centriole formation in various species would be important in the future.
